# Malaria prevalence, knowledge, perception, preventive and treatment behavior among military in Champasak and Attapeu provinces, Lao PDR: a mixed methods study

**DOI:** 10.1186/s41182-019-0138-9

**Published:** 2019-01-25

**Authors:** Phoutnalong Vilay, Daisuke Nonaka, Phosadeth Senamonty, Malayvanh Lao, Moritoshi Iwagami, Jun Kobayashi, Paul Michael Hernandez, Ketkesone Phrasisombath, Sengchanh Kounnavong, Bouasy Hongvanthong, Paul T. Brey, Shigeyuki Kano

**Affiliations:** 1 Department of Global Health, Graduate School of Health Sciences, University of the Ryukyus, 207 Uehara-cho, Okinawa, 903-0215 Japan; 2 Center of Malariology, Parasitology and Entomology, Ministry of Health, Vientiane, Lao PDR; 3 SATREPS Project for Parasitic Diseases, Vientiane, Lao PDR; 4 Military Institute of Diseases Prevention, Department of Military Medical, Ministry of Defense, Vientiane, Lao PDR; 5 Department of Tropical Medicine and Malaria, Research Institute, National Center for Global Health and Medicine, 1-21-1 Toyama, Shinjuku-ku, Tokyo, 162-8655 Japan; 6 Institut Pasteur du Laos, Ministry of Health, Vientiane, Lao PDR; 7 Department of Environmental and Occupational Health, College of Public Health, University of the Philippines Manila, 625 Pedro Gil Street, Ermita, 1000 Manila, Philippines; 8 Academic Affairs Division, University of Health Sciences, Vientiane, Lao PDR; 9 Lao Tropical and Public Health Institute, Ministry of Health, Vientiane, Lao PDR

**Keywords:** Malaria, Military, Prevalence, Knowledge, Perception, Preventive measure, Treatment behavior, Laos

## Abstract

**Background:**

Malaria is a major health problem in Lao People’s Democratic Republic (Lao PDR) with high transmission in remote and forest areas, particularly in the South. The military is at risk of malaria infection especially those deployed in forest areas. This study determined the prevalence of malaria infection and assessed knowledge, perception, and preventive and treatment behavior regarding malaria among military personnel in two southern provinces in Lao PDR.

**Methods:**

Quantitative and qualitative approaches were undertaken in Champasak and Attapeu provinces in 2017. From 313 military personnel, quantitative data were collected through questionnaire-based interviews and blood samples used for parasite detection by polymerase chain reaction (PCR). Qualitative data were collected through 7 focus group discussions and 17 in-depth interviews among 49 military personnel. Fisher’s exact test and Mann-Whitney *U* test were used to assess the association between malaria infection and participant characteristics. Content analysis for qualitative data was performed to explore perception and treatment behaviors regarding malaria.

**Results:**

The prevalence of malaria infection was 11.2% (*Plasmodium falciparum*: 1.3%, *Plasmodium vivax*: 9.3% and mixed infections: 0.6%). Many participants understood that malaria is transmitted through mosquito bites, although they did not necessarily know the name of vector mosquitoes (*Anopheles*). Surprisingly, more than a half also believed that malaria is transmitted through drinking stream water. One-third of the participants used long-lasting insecticidal nets. Due to limited supply, participants were often unable to use mosquito repellent and coils when necessary. Because participants were unable to receive timely diagnosis and appropriate treatment for malaria in their camps, they commonly practiced self-treatment using antibiotics, painkillers, and/or traditional medicines. They only go to a healthcare facility through their supervisor if their conditions worsen.

**Conclusions:**

The prevalence of symptomatic and asymptomatic malaria was conspicuous among military in forest areas. Many participants believed that malaria is transmitted not only by mosquito bites but also from drinking stream water. Preventive equipment was often insufficient. Self-treatment was practiced before referring to healthcare facility. To further prevent military from contracting malaria, the National Malaria Control Program and military body should provide adequate and suitable health education, protective equipment, and on-site malaria case management.

## Introduction

In Lao People’s Democratic Republic (Lao PDR), malaria transmission is low in plains or high-altitude are as, and high in remote, hilly and forested areas particularly in the south [1]. Although the provision of long-lasting insecticide nets (LLINs) at highly subsidized price, and free screening and case management with artemisinin-based combination therapy have aided in reducing the incidence of malaria, malaria remains to be a serious public health problem in the southern part of Lao PDR [2]. Approximately 94% of malaria cases (34,083/36,115) were reported from endemic areas in the south including Savannakhet, Saravanh, Sekong, Champasak, and Attapeu provinces in 2015. According to the malaria surveillance information, approximately 57.7% of the cases was *Plasmodium vivax*, followed by *Plasmodium falciparum* (40%) and mixed infection of *P*. *falciparum* and *P*. *vivax* (2.3%) in 2015. In the same year, malaria mortality rate was 0.03 deaths per 100,000. However, malaria morbidity rate, defined as annual parasite incidence which is the number of cases per 1000 population, was 4.9 based on the progress report for Lao PDR’s Sustainable Development Goal 3.3.4a [3]. Recently, a study showed that asymptomatic malaria is a concern in Southeast Asia [4]. National malaria surveillance systems in this region rely on passive case detection that could not investigate asymptomatic malaria [5]. On the other hand, two province-wide community-based surveys targeting villagers with or without symptoms revealed that at least 95% of the cases were asymptomatic malaria [6, 7]. Common cases reported are among adults working or living in the forest areas to seek for the source of their livelihood [8]. Due to both the terrain and remoteness, most cases involve farmers and forest workers exposing them to outdoor biting vectors [9].

The military is one of the mobile population risk groups that work in forest and border areas where transmission intensity of malaria is high [10–12]. They comprise 2 to 3% of the annual malaria cases in Lao PDR. A community-based study was conducted in six villages in Attapeu province which showed that soldiers are significantly more likely to have malaria infection, compared to other villagers [6]. Studies that were conducted with the military in other countries reported that diagnosis and treatment are often delayed because of barriers to accessing health services which can contribute to prolonging infectivity, increasing drug resistance, and promoting diseases transmission [13, 14]. No study has been conducted in Lao PDR which explores the burden of malaria and preventive and treatment behavior among military personnel. Therefore, information is lacking to guide and implement effective control strategies for this risk group.

This study aimed to (1) determine prevalence of malaria infection and (2) assess knowledge, perception, and preventive and treatment behavior regarding malaria among military personnel in Champasak and Attapeu provinces in Lao PDR.

## Materials and methods

### Study design

In this study, a combination of quantitative (cross sectional study) and qualitative methods was used. The quantitative method included questionnaire-based interview, body temperature measurement, and malaria testing through rapid diagnostic test (RDT) and polymerase chain reaction (PCR) assay. The method aimed to examine knowledge, belief, preventive measures pertaining to malaria, and prevalence of infection. The qualitative method included focus group discussions (FGDs) and in-depth interviews (IDIs). The method aimed to explore perception and behavior.

### Study sites

**Fig 1:**
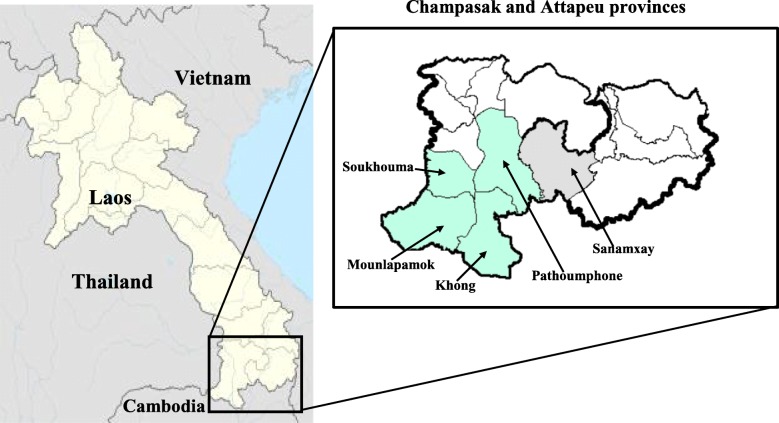
Map of the study sites in Soukhouma, Mounlapamok, Khong, Pathoumphone districts of Champasak province and Sanamxay district of Attapeu, Lao PDR

The two southernmost provinces, Champasak and Attapeu, were selected. Study districts were purposely selected by the Military Health Office using the following criteria: (1) presence of military camps in the national borders, and (2) high endemicity of malaria. These were Pathoumphone, Khong, Soukhouma, and Mounlapamok (four of ten) districts of Champasak province and Sanamxay (one of five) district of Attapeu province (Fig. 1). All the nine military camps in these districts were included in the study. Fieldwork was done in September while laboratory works were conducted in October 2017.

### Study participants

A month prior to data collection, an invitation notice was posted in the target military camps inviting military personnel to join the study. Those who came on the day of data collection were oriented about the study including the inclusion criteria and data collection procedures, and that the military personnel were free to participate in the study. Military personnel who worked in the camps are at least 18 years old, and gave consent to underwent quantitative data collection. Out of the 351 military personnel, 313 were eligible for the study. Three hundred thirteen participants in quantitative study were asked to join in qualitative data collection referring to malaria episode. Among those, only 49 participants were able to wait and voluntarily join in either FGDs or IDIs.

### Quantitative data collection

Five surveyors (one medical officer from the health sector and four medical officers from the military sector) were recruited for data collection. They were explained about the study objectives and underwent training on data collection procedures, blood sampling method, and ethical issues. The survey questionnaire included closed- and open-ended questions and consisted of four sections. The background characteristics section included questions on age, marital status, ethnic group affiliation, education level, monthly income, duration of work in remote forest areas, and previous malaria episodes in the past 1 year which obtained from all self-reported malaria. The knowledge section included questions on symptoms, transmission route, vector and breeding site, and preventive measures. The prevention section asked about routine preventive practices when participants work in forest areas. The beliefs section was on beliefs of malaria such as malaria being preventable, curable, possible cause of illness, or death. The questionnaire was pilot-tested among five military personnel in Vientiane, the capital city of Lao PDR. Minor modifications of the questionnaire were made to ensure that the words used were understandable and acceptable.

The interviews were conducted in a place where participants felt relaxed and confident to openly discuss the interview questions, mostly outside their camps. The interviews lasted about 40 to 45 min. Twenty-five interviews were excluded due to incompleteness of the answers. The remaining 288 (92%) were included in the analysis. Only 274 out of the 288 respondents were assessed for knowledge as 14 participants did not know about malaria.

### Plasmodium infection determination

Two surveyors and three to four medical personnel from their respective camps assisted in *Plasmodium* infection determination. After the questionnaire-based interview, temperature was taken and 0.2-ml blood samples were collected from the participants. Two diagnostic methods were used to detect *Plasmodium* infections in blood samples. The participants were detected on-site for malaria using RDT (SD Bioline Ag Pf/Pv, Standard Diagnostics, Inc., Gyeonggi-do, Republic of Korea). Blood samples were collected on filter papers (Whatman FTA Classic Cards, GE Health care Life Science, UK) for PCR analysis. All blood samples were analyzed using PCR method performed in Institut Pasteur du Laos. Asymptomatic infection was defined as *Plasmodium* infection detected in participants who presented with body temperature ≤ 37.5 °C and absence of any clinical symptoms of malaria at the time of blood sampling [15, 16], but tested positive by PCR. Those who had fever or tested positive in RDT during data collection was either referred or brought to the military hospital. The names of those who tested positive in PCR were forwarded to the Military Health Office which facilitated treatment for them.

#### Laboratory procedures

In accordance with the manufacturer’s instructions, deoxyribonucleic acid (DNA) was extracted from dried blood spot on the filter papers with a QLAamp DNA Mini Kit (Qiagen, Hilden, Germany). The extracted DNA was eluted with 50 μL of elution buffer in the kit and preserved at − 30 °C. A real-time nested PCR was performed using a primer set to identify malaria parasite infection [17, 18]. A universal primer set was use in the primary real-time PCR to amplify the partial *cytochrome b* gene on the mitochondrial genome of malaria parasites. In the secondary real-time PCR, *P*. *falciparum* and *P*. *vivax* were detected using specific primer sets. The real-time PCR was performed using SsoAdvanced™ Universal SYBR® Green Supermix (Bio-Rad Laboratory, Inc., USA) using 2 μL of the extracted DNA as a template, which was equivalent to 1.2 to 1.6 μL of whole blood. The primary PCR product was diluted 25 times with PCR-grade water, and 2 μL of the diluted primary PCR product was used as a template for the secondary real-time PCR. Serial diluted recombinant plasmid DNAs containing the *cytochrome b* region of *P*. *falciparum* and *P*. *vivax* were used as positive control for each assay, and PCR-grade water was used as negative control. A sample was considered negative if there was no indicated line increase in the SYBR® Green (fluorescent) signal after 35 cycles. When positive results were obtained at least twice, the sample was considered positive for *Plasmodium* DNA [6].

### Qualitative data collection

After the questionnaire-based interview, blood sampling, and temperature measurements, participants were invited to participate in IDI or FGD in the same or the following day. IDIs and FGDs were conducted in Lao language. The IDI and FGD guides comprise of questions related to participants’ views about malaria, risks related to their living and working conditions, information about their health, their care-seeking and treatment behavior when getting malaria, preventive measure use for malaria in the forest, and sources of malaria information. Proceedings were recorded using a digital voice recorder with the participants’ permission. Most of the FGDs and IDIs were carried out outside their camps. Immediately after the interviews, all were invited and selected referring to malaria episode. Those who gave consent had the option to participate in either FGD or IDI. Four to five participants joined the FGDs while 17 participants participated in IDIs. Seven FGDs with 32 participants and 17 IDIs were performed. The FGDs and IDIs lasted between 35 and 50 min.

### Quantitative data analysis

Data were double entered into Microsoft Excel version 2016. Incorrect entries were examined and verified against the original forms. The infection of malaria, prevalence, knowledge, and believe regarding malaria were analyzed and presented using frequencies. The participants’ age, monthly income, and duration of work in the current military camp were treated as continuous variables and summarized as medians with interquartile range (IQR). Proportion and 95% confidence interval (CI) of military infected with malaria using PCR was calculated. Fisher’s exact test and Mann-Whitney *U* test were used to assess the associations between malaria infection status determined by PCR assay and respondent’s general information (age, gender, ethnicity, education, marital status, monthly income, and duration of work in the current military camp) and use of a prevention measure. A *p* value < 0.05 was considered statistically significant. These analyses were performed by SPSS version 23.

### Qualitative data analysis

Transcriptions of the IDIs and FGDs were done by the first author (PV). The transcripts were analyzed using content analysis, which is a stepwise analytical process, focusing on description and interpretation of underlying meanings of the text [19]. In order to minimize misinterpretations, interview notes were used side-by-side. Words, phrases, and sentences as a part of text in the transcript were used to develop codes. The codes that reflected the core meanings of the interview text were identified, and grouped into subcategories, categories, and themes. Data analysis was supervised and validated by the sixth author (JK) who is an expert on qualitative data analysis.

## Results

### Background characteristics of the participants

The median age of participants was 28 years and median work duration in forest areas was 4 years (Table 1). Majority of the participants (98.6%) were men. The educational attainment was mostly high school or lower (81.9%). Approximately less than half (45.5%) of the participants were married. Approximately one-third (33.7%) reported to experience a malaria episode at least once in the past year. Among almost all the participants (98.3%), their body temperatures were equal to or less than 37.5 °C during the survey. Among the 35 participants who tested positive by PCR, 34 (97.1%) did not have a fever greater than 37.5 °C.

**Table 1:** Characteristics of military personnel

Characteristics	Total		Positive		Negative		*p* value
	*n* = 288	%	*n* = 35	%	*n* = 253	%	
Age (years), median (IQR)	28 (24 to 34)		27 (24 to 40)		28 (24 to 34)		0.455
Monthly income (USD), median (IQR)	151.9 (102.3 to 188.4)		151.9 (99.4 to 187.0)		151.9 (102.3 to 188.4)		0.593
Work in forest areas (years), median (IQR)	4 (2 to 7)		5 (2 to 10)		3 (2 to 7)		0.030
Gender							
Male	284	98.6	35	100.0	249	98.6	1.000
Female	4	1.4	0	0.0	4	1.4	
Ethnicity							
Lao ethnic	252	87.5	29	82.9	223	88.1	0.411
Other ethnic	36	12.5	6	17.1	30	11.9	
Education							
≤ High school	236	81.9	29	82.9	207	81.8	1.000
> High school	52	18.1	6	17.1	46	18.2	
Marital status							
Single	157	54.5	22	62.9	135	53.4	0.366
Married	131	45.5	13	37.1	118	46.6	
Episode of malaria (times)							
Never	191	66.3	21	60.0	170	67.2	0.671
1 or more	97	33.7	14	40.0	83	32.8	
Body temperature (°C)							
≤ 37.5	283	98.3	34	97.1	249	98.4	0.479
> 37.5	5	1.7	1	2.9	4	1.6	
Provinces							
Champasak	227	78.8	28	80.0	199	78.7	0.528
Attapeu	61	21.2	7	20.0	54	21.3	

When comparing these characteristics between the positive and negative groups as detected by PCR, the working duration was significantly longer in the positive group than in the negative group (*p* = 0.030). For other characteristics, there were no significant differences between the two.

### Prevalence of malaria infection

PCRs detected malaria parasitemia in 35 of 313 blood samples (11.2%, 95% CI 7.7 to 14.7) (Table 2). The species distribution was *P*. *falciparum* mono-infection at 1.3% (4/313), *P*. *vivax* mono-infection at 9.3% (29/313), and *P*. *falciparum* and *P*. *vivax* mixed infections at 0.6% (2/313). RDTs identified only three *P*. *vivax* mono-infection, six *P*. *falciparum* mono-infection, and one *P*. *falciparum* and *P*. *vivax* mixed infection.

**Table 2:** Distribution of malaria infection according to species among those who tested positive by PCR (*n* = 35) and RDT (*n* = 10)

Type of malaria infection	PCR	RDT
	*n* (%)	*n* (%)
*P*. *vivax* mono-infection	29 (82.9)	3 (30.0)
*P*. *falciparum* mono-infection	4 (11.4)	6 (60.0)
*P*. *falciparum* and *P*. *vivax* mixed-infection	2 (5.7)	1 (10.0)

### Knowledge on malaria

Only 47.4% of the participants knew that malaria is transmitted by an *Anopheles* mosquito bite (Table 3). The participants believed that malaria is transmitted by drinking stream water from the forest (60.9%). Fewer participants also believed that malaria is transmitted through coughing/sneezing (28.5%) or by flies (25.5%). In contrast, participants knew about abnormal health conditions that are considered malaria symptoms: more than 80% of the participants knew that fever, headache, body pains, and chills are symptoms of malaria.

**Table 3:** Knowledge on transmission routes and symptoms of malaria among study participants (*n* = 274)

Knowledge	Yes	No	Do not know
	*n* (%)	*n* (%)	*n* (%)
Malaria transmission route			
Anopheles mosquito biting	130 (47.4)	29 (10.6)	115 (42.0)
Drinking stream water in forest	167 (60.9)	46 (16.8)	61 (22.3)
Cough or sneeze	78 (28.5)	102 (37.2)	94 (34.3)
Flies	70 (25.5)	100 (36.5)	104 (38.0)
Spirit	19 (6.9)	172 (62.8)	115 (42.0)
Malaria symptoms			
Fever	242 (88.3)	7 (2.6)	25 (9.1)
Headache	236 (86.1)	9 (3.3)	29 (10.6)
Body pains	235 (85.8)	7 (2.6)	32 (11.7)
Chills	234 (85.4)	10 (3.6)	30 (10.9)
Weak/poor appetite	173 (63.1)	51 (18.6)	50 (18.2)
Vomiting	163 (59.5)	44 (16.1)	67 (24.5)

### Beliefs about malaria

Most of the participants (93.8%) showed fear of contracting malaria (Table 4). Most of the participants (92.0%) believed that working in the forest puts them at risk of acquiring malaria. More than 80% of participants also believed that malaria is preventable, curable, but also can cause illness and death.

**Table 4:** Beliefs on malaria infection among study participants (*n* = 288)

	Yes	No	Do not know
	*n* (%)	*n* (%)	*n* (%)
Fear of getting malaria	270 (93.8)	11 (3.8)	7 (2.4)
Working in forest areas is risk acquiring malaria	265 (92.0)	8 (2.8)	15 (5.2)
Malaria is preventable	260 (90.3)	4 (1.4)	24 (8.3)
Malaria is curable	256 (88.9)	5 (1.7)	27 (9.4)
Malaria causes of illness	256 (88.9)	11 (3.8)	21 (7.3)
Malaria can cause of death	242 (84.0)	13 (4.5)	33 (11.5)

### Preventive measures

Almost all participants (99.0%) used a bed net (Table 5). Some of them (*n* = 80) owned LLIN. A few numbers of participants (*n* = 31) used hammock nets. Most of the participants (91.3%) used mosquito repellent. Fewer participants used wood/plant smoke (68.4%) and mosquito coil (87.8%) when they stayed in a forest area. About two-thirds of the participants (73.6%) took drug prophylaxis distributed in their camps. When comparing these preventive measures between positive and negative groups, drug prophylaxis was significantly associated with malaria infection (*p* = 0.039). There were no statistically significant associations observed between malaria infection and the other preventive measures.

**Table 5:** Preventive measure of malaria among study participants (*n* = 288)

Prevention practices	Total *n* = 288	Positive *n* = 35	Negative *n* = 253	*p* value^a^
	*n* (%)	*n* (%)	*n* (%)	
Bed net use				1.000
Yes	285 (99.0)	35 (100.0)	250 (98.8)	
No	3 (1.0)	0 (0.0)	3 (1.2)	
Ordinary bed net				0.837
Yes	215 (74.7)	27 (77.1)	188 (74.3)	
No	73 (25.3)	8 (22.9)	65 (25.7)	
LLINs				0.226
Yes	80 (27.8)	13 (37.1)	67 (26.5)	
No	208 (72.2)	22 (62.9)	186 (73.5)	
Hammock net				1.000
Yes	31 (10.8)	3 (8.6)	28 (11.1)	
No	257 (89.2)	32 (91.4)	225 (88.9)	
Wearing long uniform				0.713
Yes	269 (93.4)	32 (91.4)	237 (93.7)	
No	19 (6.6)	3 (8.6)	16 (6.3)	
Repellents use				0.750
Yes	263 (91.3)	33 (94.3)	230 (90.9)	
No	25 (8.7)	2 (5.7)	23 (9.1)	
Mosquito coil use				0.782
Yes	253 (87.8)	32 (31.4)	221 (87.4)	
No	35 (12.2)	3 (8.6)	32 (12.6)	
Wood/plant smoke use				0.702
Yes	197 (68.4)	23 (65.7)	174 (68.8)	
No	91 (31.6)	12 (34.3)	79 (31.2)	
Taking drug prophylaxis				0.039
Yes	212 (73.6)	31 (88.6)	181 (71.5)	
No	76 (26.4)	4 (11.6)	72 (28.5)	

^a^Fisher’s exact test

### Content analysis on qualitative data

The findings are presented according to the five themes that have emerged: (1) direct and indirect effects of malaria, (2) perception on malaria, (3) self-care, (4) health service delivery, and (5) access and use of preventive equipment (Table 6).

**Table 6:** Themes, categories, and sub-categories structure

Themes	Categories	Sub-categories
1. Direct and indirect effects of malaria	Common health problem	Dengue fever Malaria episode Health problems in the family Other diseases
2. Perception of malaria	Information sources Lack of knowledge Understanding of malaria	Dangerous areas Cause of malaria Fear of malaria infection
3. Self-care	Preventive behaviors Purchase of personal preventive tools Self-medication Seeking care	Preventive measures Good practice Emphasizing on repellent and LLIN use
4. Health service delivery	Process of making decision Health care services	Field services Referral patients Health facilities which hey access when they get sick
5. Access and use of preventive equipment	Insufficiency of preventive tools Lack of diagnostic kits and anti-malaria Difficulty in accessing health facilities Difficulty in using bed nets Exposure to malaria	

#### Direct and indirect effects of malaria

Malaria was recognized as the second most common health problem next to dengue fever. Majority of the participants recognized that *P*. *vivax* is common among military personnel and that malaria is a cause of their hospitalization. Upon hospitalization of the respondents or their colleagues, they ask the medical personnel about their sickness hence they are able to identify the cause of their condition. They have also observed the symptoms among their sick colleagues. This information is shared in their camps. Participants commented that it is not easy to protect themselves from malaria infection. One participant who had the infection narrated how this occurred.

The common disease among the military is malaria. I cannot avoid getting infected. In 2014, the doctor told me that I got mixed-infection with *P*. *falciparum* and *P*. *vivax*, then I got the infection again in 2015 and 2016 with *P*. *falciparum*, and in 2017 with *P*. *vivax*. [22–32-year-old military, in FGD 3, Khong district].

Not only themselves but also their family members suffered from malaria. Participants who were living with their family members reported that malaria affected many aspects of their lives, especially on finances. Despite that the malaria treatment is free at hospitals, participants said that expenses for medicines other than antimalarials, and other daily medical expenses are not fully covered. Therefore, many participants spent their families’ money or needed to borrow money from their relatives.

#### Perception of malaria

The main sources of information about malaria were television, colleagues in their camp, and the people in the nearby village. Many participants are concerned since others in the military community have been seriously infected with malaria. Additionally, they have expressed concern that they were working in malaria high-risk areas. It was hard for the participants to differentiate malaria and dengue fever as the symptoms of these diseases were similar. Many explained that malaria transmission was through a mosquito bite. Among those who were infected, they confidently said that an *Anopheles* mosquito bite is the cause. Many participants believed that being weak or not having enough rest make them at risk of contracting malaria. Some said that people with good physical conditions or a healthy person could not have malaria. Some participants believe that drinking unclean water from breeding sites is the cause, especially if it is contaminated with mosquito eggs, as this military elaborated:

There are many streams and ponds where mosquito reproduction happens. When we drink water, especially if it is contaminated with mosquito eggs, then we get infected. [22–33-year-old, military in FGD 1, Pathoumphone district].

#### Self-care

Most of the participants relied on preventive tools such as ordinary bed nets, mosquito repellents, and mosquito coils to protect themselves from mosquitoes. Some of them had used LLINs or insecticide-treated bed nets (ITNs). They emphasized that LLINs and repellents are necessary and there should not be any shortage for these in military camps in forests. In addition, some of the participants had to buy their own preventive tools at local markets. Chloroquine (CQ) was distributed for malaria prophylaxis only in Champasak province.

Participants noted that self-medication was their first response in the forest because of the limited access to treatment services. Antibiotics and painkillers were commonly used before referring a patient to a healthcare facility, but for some, traditional/herbal medicine was used as an alternative. Most of the participants reported that they used medicines that they brought or provided by the camp. If they do not feel better in 2 to 3 days, they would consult the health advisor or request a colleague to help them to seek medical care. One young man explained his ill-health condition:

I had fever in the forest and I was recommended not to take anti-malaria pills as it will influence the results of the diagnostic test. I took the painkillers like Paracetamol and just waited. It took me two to three days from the hilly forest area to meet the camp health advisor. I was then sent to a health center. [28-year-old, military, IDI 17, Sanamxay district].

#### Health service delivery

Based on the participant’s discussions, military body and the military medical team have established healthcare services. General healthcare services and essential medicines are provided to the military personnel except malaria test kits and antimalarial medicine. In addition, a referral system has been launched to provide early advanced medical care. Although the healthcare services are in place, these are not fully established in all camps. When a patient has to be referred, some had to wait for 2 days to a week. There were no on-site malaria diagnostic and treatment services, despite that they have to work 2 to 3 months in the remote forest areas. When somebody is ill, the existing healthcare services are used. Almost all participants said that they first seek consult to their camp health advisor but the decision to refer the military manifesting clinical symptoms to a healthcare facility is made by the military team in which the ill patient is a member. Those who had not sought treatment said that they live very far from health facilities or they did not know where to get treatment. Those who were referred to community health centres and district hospitals said that there are enough medical supplies in these facilities.

#### Access and use of protective equipment

Basic preventive tools such as ordinary bed net, repellents, and mosquito coils were distributed free of charge in the camps. In addition, LLINs came from local health offices. However, there is still a lack of preventive equipment which limited the participants’ ability to protect themselves against mosquitoes. Some participants used repellents, anti-mosquito lotions, coils and hammock nets, or burned fire wood to create smoke while doing outreach inspection outside the camps at night. Inadequate distribution of LLINs emerged in some focus groups. One military personnel said:

We received LLINs in 2016 but only two nets for our group were provided. Only two persons can use it at a time. We need nets for everyone in the camp. [22–33-year-old, military in FGD 1, Pathoumphone district].

Several participants said that while traveling in the evening, when mosquitoes are active, they do not to protect themselves from mosquito bites as there is a lack of space in the forest to hang the bed nets, difficulty in using these particularly during the rainy season, and inability to use these during security inspections. It was often said that participants had to buy preventive tools at local markets. Participants also argued that not only preventive tools should be provided. There should also be on-site diagnostics tests and antimalaria drugs. They added that they had to wait for several days for them to be treated in the camps. In addition, drug prophylaxis for malaria was also requested from the interviewer.

## Discussion

In the present study, the prevalence of malaria infection determined by PCR was 11.2% (35/313). Most of the infections (82.9%) were *P*. *vivax* mono-infections and almost all the infections were asymptomatic (97.1%). The finding suggests that the military in the study sites are at risk of *P*. *vivax* infection. This finding is similar to that from a community-based study conducted in the endemic villages in three districts of Attapeu province including Sanamxay district where the present study was also conducted. The study reported that the prevalence of malaria infection was 6.6%, and *P*. *vivax* mono-infection and asymptomatic infection account for 87.2% and 97.9% of the total infection, respectively. Evidence suggests that individuals with asymptomatic, low-density malaria infection can contribute to local transmission [6, 20]. Therefore, without addressing malaria among the military in southern provinces, it would be impossible to achieve the target of Lao National Malaria Control and Elimination Program: eliminating malaria in the country by 2030.

Most of the infections were due to *P*. *vivax* as revealed in the present study that was similar to a study among communities in Nong district, Savannakhet province, Lao PDR [7]. It requires to have a test of glucose-6-phosphate dehydrogenase (G6PD) deficiency before treatment of each *vivax* malaria patient by primaquine therapy. G6PD test and primaquine is available in provincial and district hospitals [6]. A 14-day course of primaquine was recommended to complete radical cure of *vivax* malaria, but this drug can cause a serious side effect, e.g., from mild to severe hemolysis in patients with glucose-6-phosphate dehydrogenase (G6PD) deficiency [21]. The patients that were infected by *vivax* malaria had moderate levels of serious G6PD deficiency among Lao population was reported [22]. Most of the vivax infection patients reside in remote areas, which are far from the hospitals. Additionally, health centers are not allowed to prescribe primaquine (as indicated in the guidelines) [6]. The limited access to G6PD test and primaquine in rural areas may contribute to delaying elimination of malaria. These problems can only be addressed by improving the policies, guidelines, and the facilities.

In the present study, PCR was used to identify four falciparum mono-infections, whereas RDT was used to identify six falciparum mono-infections. The difference might be due to false positive results of RDT, because the sensitivity and specificity of PCR for *P*. *falciparum* is higher than those of RDT [23], and because the histidine rich protein 2 (HRP-2), which is a target antigen for detecting *P*. *falciparum*, can persist for 28 days in peripheral blood even after effective treatment [24].

The present study showed that, although most participants understood the connection between malaria and mosquito, they did not necessarily know the name of malaria vector mosquitoes (i.e., *Anopheles*). The fact that people in a malaria endemic area understand the connection between malaria and mosquito was also reported in a recent Lao study that was conducted with general population in Nong district: The study reported that 96.8% of the participants understood the connection between malaria and mosquito [25].

Adequate knowledge and perception of malaria is important for its prevention [26, 27]. It also allows timely diagnosis and improves treatment seeking behavior [28]. The participants of the present study acknowledged the connection between the forest and malaria. However, many participants believed that drinking stream water in forests can cause malaria. A study that were conducted in Nong district showed the villagers described proper knowledge of the prominent malaria symptom, such as fever with chills and beliefs that malaria is a cause of severe illness and death [29]. The finding of the present study was consistent with that of the Nong study. The participants of the present study also believed that not only mosquito bites but also drinking unclean water can transmit malaria. This finding is also compliant with the Nong studies [25, 29]. These studies show that villagers make a link between malaria and poor hygiene, as well as via a mosquito bite. Additionally, the participants perceived that having good physical conditions or being healthy protects them from contracting malaria. Also, this result was similar with the Nong district study which revealed that some believe that cleaner persons will not have malaria [29]. This erroneous perception of malaria transmission may lead to incorrect preventive practices. For instance, a study in northern Ghana showed that people who have limited malaria knowledge including the role of vector were less likely to use ITNs [30]. In Nigeria, caregivers of children under-five who did not know what causes malaria and did not know methods to prevent malaria were less likely to use ITNs, even though they owned one [31]. Providing health education to the military community can promote correct understanding of malaria transmission route. It can also enhance their health concern, treatment-seeking behavior, and preventive practices. This would help them improve preventive practices for malaria. Furthermore, the participants who were dispatched to high-risk areas of malaria infection should know malaria, especially its symptoms. This allows them to recognize malaria infection and seek early treatment.

The study participants used a variety of preventive measures to prevent mosquito bites and three-fourths of them owned ordinary bed nets. A study in a neighboring country found out that even if untreated bed net could prevent malaria in village community, it was not suitable for persons who work in forest areas [32]. A study in India showed that malaria prevalence among people who used insecticide-treated nets was lower than those who used untreated bed net in low endemic and high endemic areas [33]. Moreover, shifting malaria prevention activities to the use of LLINs significantly reduced malaria prevalence [34]. Also, in Lao PDR, malaria intervention activities which includes LLINs distribution to general population at risk succeeded in reducing the number of malaria cases by 92% in 2010 [2]. Evidence suggests that if LLINs would be widely distributed to military, then malaria prevalence among those assigned in forest areas would become lower.

When the participants worked and traveled overnight in the forest, they had some difficulty in using bed nets because of inappropriate location and they do not have proper sleeping areas. Among the participants, hammock net was familiar and suitable for them. However, only 10.8% of the participants owned a hammock net. Additionally, those who did not have hammock net consequently burned firewood to produce smoke that will prevent mosquito bites at night. In the Cambodian and Vietnam border, hammock net was popularly used among young and adult men when sleeping overnight outside their homes [35]. In the same manner, long-lasting insecticide hammock nets (LLIHs) were effective in reducing malaria vector bites and protecting forest workers in Cambodia [36]. Furthermore, LLIH was identified to be a feasible method in reducing the incidence of malaria in forest areas [37]. The participants of the present study often worked and slept outside their camps in the forest. Hence, LLIHs should be an additional preventive tool for the military who work for security inspections in Lao PDR.

At night, while on security inspection and before going to sleep, participants did not use bed nets. Furthermore, the results of the qualitative analysis revealed that mosquito repellent was often unavailable when necessary. This was because the military authority provided an inadequate supply of repellent lotion to the staff. The results of a systematic review showed that high level of protection by repellent was effective against *Anopheles gambiae s.l.* in the field in Tanzania. Moreover, repellents were effective in reducing malaria morbidity [38]. Also, distribution of highly effective repellents could prevent malaria infection [39]. Improvement and ensuring adequate repellent supply to military employed in forest areas could solve the issue of mosquito biting when the participants work during the active hours of the mosquito vectors.

The present study found that taking drug prophylaxis was significantly associated with malaria infection status. The association is likely to be confounded by third factors; i.e., factors other than the drug prophylaxis and malaria infection status. Especially the association could be due to confounding by indication. That is, participants for whom drug prophylaxis was prescribed are often at higher risk of malaria infection, compared to those for whom drug prophylaxis was not prescribed. Thus, participants who reported taking drug prophylaxis showed higher prevalence, compared to those who did not.

The present study showed that taking chloroquine prophylaxis was common among study participants. More attention should be paid to this practice, because inappropriate use of chloroquine can promote chloroquine resistance [40]. Although the present study did not examine how participants administered prophylaxis, there is a possibility of sub-optimal use of chloroquine among participants, as reported by a study in Africa [41]. Additionally, non-compliance with antimalarial drug regimen or receiving sub-optimal dose led to increased risk of treatment failure [42]. Poor adherence to antimalarial medication for uncomplicated malaria was observed among rural communities in Lao PDR [43]. Failure to monitor this concern would affect prevalence of antimalarial drug resistance [44].

Case management of malaria in military health service at forest areas was not effective. Recently, an initial cooperation between the military organization and the National Malaria Control Program (NMCP) have begun which included training military doctors and medics with the current national guidelines such as case management and prevention strategies [10]. However, case management procedures should be improved. These procedures should be aligned with the national guidelines on malaria treatment. There should be appropriate diagnostic tool kits and antimalarial drug combination therapy (ACTs) as current first-line treatment as introduced by the National Program [45, 46] to improve early access to malaria diagnosis and treatment [15]. The NMCP should assist the medical teams in the military to ensure adequate supply of malaria diagnosis tools (RDTs) and ACTs [47] and skilled military healthcare workers through training on case management. The program should also assist in monitoring these interventions among the military. Malaria mobile teams or malaria posts were very helpful to reduce malaria prevalence among villages in remote areas in neighboring countries [48, 49]. A regional malaria elimination program that was implemented in Eastern Myanmar showed that providing early diagnosis and effective treatment through community-based malaria posts substantially decreased village-level incidence of malaria [50]. It is possible to train camp military medical or military personnel and participate in malaria posts. They can cover catchment areas to improve early diagnosis and treatment.

The present study discovered some difficulty of receiving malaria diagnosis and treatment at the health facilities due to the lack of medical personnel and inadequate health facilities. There is a need to improve accessibility to healthcare services for malaria treatment in remote communities [29]. In addition, poor accessibility to health facilities by limited transportation and high-hilly road condition was a common problem in isolated remote villages preventing residents to receive timely malaria treatment [51].

The results identified major challenges in malaria control such as the lack of health information particularly about the causes and transmission route. The delay in seeking treatment may be due to the very remote location of the camps in hilly forests making self-medication as the best alternative among the participants [52]. Poor access to healthcare services for malaria treatment was due to long distance to the health facility as well as a high cost of transportation and referral [53]. The unavailability of RDTs and ACTs, and inadequate supply of appropriate protection tools such LLINs, repellents, anti-mosquito lotions, and coils is also a major challenge. On the other hand, the government provided intermittent and limited protective tools to this at risk population [54]. However, improving the cooperation with the Ministry of Health especially for malaria control is crucial to provide appropriate and updated information, technical support, and medical equipment. Other government agencies, such as the Ministry of Finance and Ministry of Welfare, should assist the military body in implementing sustainable interventions.

Data were only collected from military personnel who were available on the day of interview. The information of those who were absent or were in a health facility was not covered. Therefore, the present study might underestimate the prevalence of malaria infection. The camps recruited in the study were not randomly selected, and thus, the generalizability of this study’s findings can be limited. Although the data collection assistants were trained, they were military technical medical officers. This may lead to an interviewer bias since the assistant may want to avoid a negative social image. The proportion of participants who knew the name of vector mosquitoes (*Anopheles*) might be overestimated, because when the question was asked, “*Anopheles* mosquito” was provided as one of the response options. Despite these, this study provided important information that can be used when designing services and information campaigns for the military in this particular context.

## Conclusion

Military in forest border areas are at risk for malaria infection. The prevalence of malaria was 11.2% with 82.9% of which was *Plasmodium vivax* mono-infection. More than a half of the military in forest areas believed that malaria is transmitted by drinking stream water. Military practice self-medication using either antibiotics and painkillers or traditional medicine before referring patients to undergo malaria testing and treatment. Protective equipment such as LLINs and mosquito repellents were insufficient. There was also a lack of diagnostic and treatment services in the camps, and inaccessibility of health services. The NMCP and the military body should provide health education and widely distribute protective equipment. There should also be on-site malaria testing and case management to prevent delays in accessing appropriate medical care.

## Abbreviations

ACTs: Antimalarial drug combination therapy
FGDs: Focus group discussions
IDIs: In-depth interviews
IQR: Interquartile range
LLIH: Long-lasting insecticide hammock net
LLIN: Long-lasting insecticide-treated net
NMCP: National Malaria Control Program
PCR: Polymerase chain reaction
RDT: Rapid diagnosis test
